# Design and Preliminary Panel-Level Mechanical Validation of a Bistable SMA-Driven Module for CubeSat Reflector Applications

**DOI:** 10.3390/mi17101201

**Published:** 2026-10-17

**Authors:** Stefano Rodinò, Carmine Maletta

**Affiliations:** Department of Mechanical, Energy and Management Engineering, University of Calabria, 87036 Rende, Italy; carmine.maletta@unical.it

**Keywords:** CubeSat, panel-level mechanical switching, shape memory alloy, bistable mechanism, compliant structure, smart actuator

## Abstract

This paper presents the design and preliminary mechanical validation of a bistable switching module driven by shape memory alloy (SMA) springs for CubeSat reflector-panel applications. A pre-compressed curved compliant beam provides passive state retention, while two antagonistic SMA spring pairs trigger reversible switching. The module was sized for a 3U CubeSat reflector concept comprising nine panels and developed through finite-element analyses and staged prototyping. The numerical analyses supported preliminary geometry screening using the rise-to-thickness ratio and axial pre-compression. The actuator achieved a switching stroke slightly below 7 mm, and the integrated panel demonstrator reached an approximate edge displacement of 19 mm, compared with a simulated value of 21.3 mm, a difference of 10.8 percent. A ten-cycle sequence demonstrated short-term switching repeatability under the tested laboratory conditions. The results support actuator- and panel-level mechanical switching under laboratory conditions; they do not validate the surface accuracy or electromagnetic performance of a complete reflector.

## 1. Introduction

CubeSats have evolved from educational demonstrators into credible platforms for Earth observation, technology validation, and communication missions, but their compact form factor still imposes severe constraints on mass, stowed volume, available power, and deployment reliability [1,2]. These constraints are especially restrictive for communication payloads, where higher gain and mission-adaptive radiation characteristics must be reconciled with tight packaging margins and robust deployment. As a result, deployable and reconfigurable antenna architectures remain a persistent research theme in the small-satellite literature [3,4,5].

Among the candidate solutions, reflector-, reflectarray-, and deployable panel-based concepts are particularly attractive because they can decouple electrical aperture from launch volume. The ISARA flight demonstration established the practical relevance of deployable apertures in CubeSat missions [3], while more recent reviews have shown that antenna selection is increasingly governed not only by electromagnetic performance, but also by packaging efficiency, deployment strategy, subsystem integration, and structural complexity [1,2]. In parallel, a broad mechanics-oriented literature has advanced deployable antenna supports through planar modular mechanisms, flat reflective surfaces, cable-rib parabolic cylindrical concepts, and ring- or truss-based reflector architectures [6,7,8,9,10,11,12,13]. Collectively, these studies have substantially improved folding ratio, kinematic determinacy, structural stiffness, and surface-support accuracy, but they remain focused predominantly on deployment toward a prescribed geometry.

A more demanding objective is geometric reconfiguration after deployment. In this direction, recent mechanism studies have started to move beyond one-shot deployment toward structures capable of assuming more than one deployed shape. In particular, Tang et al. [14] proposed a deployable support truss for parabolic cylindrical antennas with shape reconfiguration, highlighting growing interest in multi-state antenna-support architectures. Related reflector-oriented work has also shown that SMA-enabled hingeless or superelastic members can support large deployable mesh reflectors and parabolic antenna concepts [15,16]. However, this emerging literature is still rooted mainly in large-aperture linkage-, truss-, or mesh-dominated systems, and leaves comparatively open the problem of compact, reversible, panel-scale reconfiguration modules compatible with CubeSat integration constraints.

Within this context, shape memory alloys (SMAs) are attractive actuation materials because they combine compactness, high force-to-weight ratio, simple electrical activation, and long-standing relevance to aerospace mechanisms [17]. SMA-enabled antenna adaptation has already been explored across multiple scales and functions, including RF switches and patch reconfiguration [18], thermally reconfigurable kirigami and pop-up antennas [19,20], polarization or frequency tuning concepts [21,22], flexible reflector-like antenna structures with beam-steering capability [23], and deployable SMA-composite antenna concepts with two-way folding–deploying behavior [24]. In parallel, SMA-based distributed morphing panels and adaptive composite systems have been investigated beyond the antenna field, reinforcing the broader feasibility of geometrically adaptive lightweight structures [25,26]. At the same time, the SMA-actuation literature has made clear that practical performance depends strongly on coupled electro-thermo-mechanical behavior, activation strategy, thermal management, and interface design [27,28]. Accordingly, SMA feasibility at the material or component level does not automatically translate into a compact and mechanically credible reconfiguration subsystem suitable for CubeSat reflector integration.

A second issue concerns state retention. Many thermally activated smart-structure concepts can demonstrate shape change, but they remain penalized when the target configuration must be maintained without continuous power input. This limitation is particularly important for CubeSat platforms, where electrical power is scarce, and actuation energy should ideally be spent only during state transitions. Bistable compliant mechanisms are therefore appealing because they can preserve two equilibrium states passively, store the required elastic energy internally, and reduce the need for continuous holding power [29,30].

Beyond compliant beams, SMA-triggered bistability can also be realized through rigid-link mechanisms with recuperative springs. Kostov et al. proposed a reciprocating piston-pump architecture based on a three-arm rocker and recuperative springs, and subsequently introduced a two-section involute cam to tailor the recuperative moment and bring the suction and discharge instability positions closer together [31,32]. These approaches provide relevant alternatives for load-dependent stroke transmission and force shaping, although their application to a compact reflector module would require dedicated kinematic synthesis and packaging assessment.

Taken together, the relevant literature can be organized into three partially overlapping strands: deployable and high-gain antenna architectures for CubeSats and small satellites [1,2,3,4,5,6,7,8,9,10,11,12,13,14,15,16], SMA-enabled geometric adaptation of antennas and morphing structures [18,19,20,21,22,23,24,25,26,27,28], and bistable compliant mechanisms for low-energy switching and passive state retention [29,30]. These strands clearly indicate the ingredients needed for compact reconfigurable space structures, but not yet a well-established subsystem-level solution at their intersection. In particular, experimentally grounded evidence remains limited for compact bistable SMA-driven modules explicitly sized for CubeSat reflector panels and validated as integrated mechanical reconfiguration subsystems rather than as isolated actuator demonstrations or fully antenna-centered platforms.

The present work addresses this gap by proposing a bistable SMA-driven reconfiguration module designed for integration into a 3 U CubeSat reflector-panel concept. The architecture combines a pre-compressed curved compliant beam, which defines the bistable energy landscape, with two antagonistic SMA spring pairs, which trigger the transition between the stable states. The contribution is twofold: it defines a mechanical switching-module design strategy under CubeSat geometric constraints and provides preliminary laboratory validation at the actuator and integrated panel-demonstrator levels through displacement, thermal-response, and short-cycle repeatability tests. The nine-panel reflector is an intended application concept, not a complete experimentally validated reflector.

The scope of the study is deliberately limited to subsystem-level mechanical validation. The paper does not claim a flight-ready antenna, full-reflector surface-accuracy validation, or electromagnetic characterization of a metallized reflector surface. Instead, it aims to establish whether the proposed switching principle is mechanically credible, compact, and sufficiently controllable to justify future electromagnetic and environmental maturation. Section 2 presents the materials and methods, Section 3 summarizes prototype development and panel-interface integration, Section 4 reports the experimental results and discussion, and Section 5 gives the conclusions.

## 2. Materials and Methods

### 2.1. CubeSat Reflector Concept and Bistable Actuator Architecture

**Figure 1:**
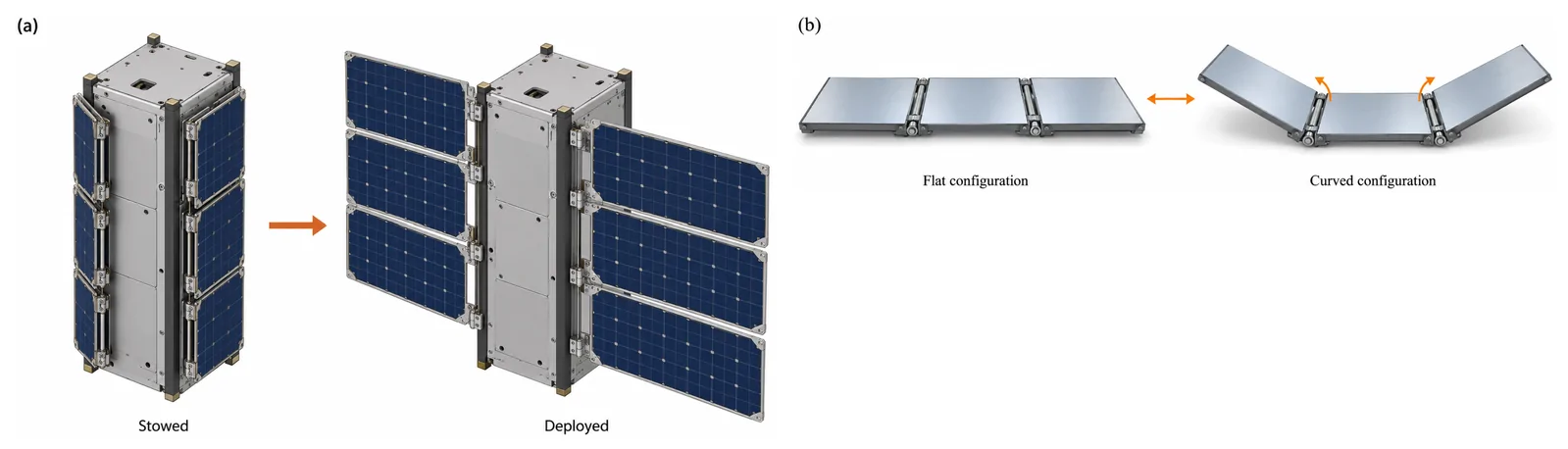
Generic conceptual illustrations of panel deployment and reconfiguration. (**a**) A CubeSat with side-mounted panels in stowed and deployed configurations. (**b**) Articulated panels in flat and faceted curved configurations. The straight arrow in (**a**) indicates deployment; the double-headed arrow in (**b**) indicates reversible switching, and the curved arrows indicate joint rotation. The illustrations are not to scale and do not represent experimental results, a validated mechanism, or specific hardware from the cited publications.

**Figure 2:**
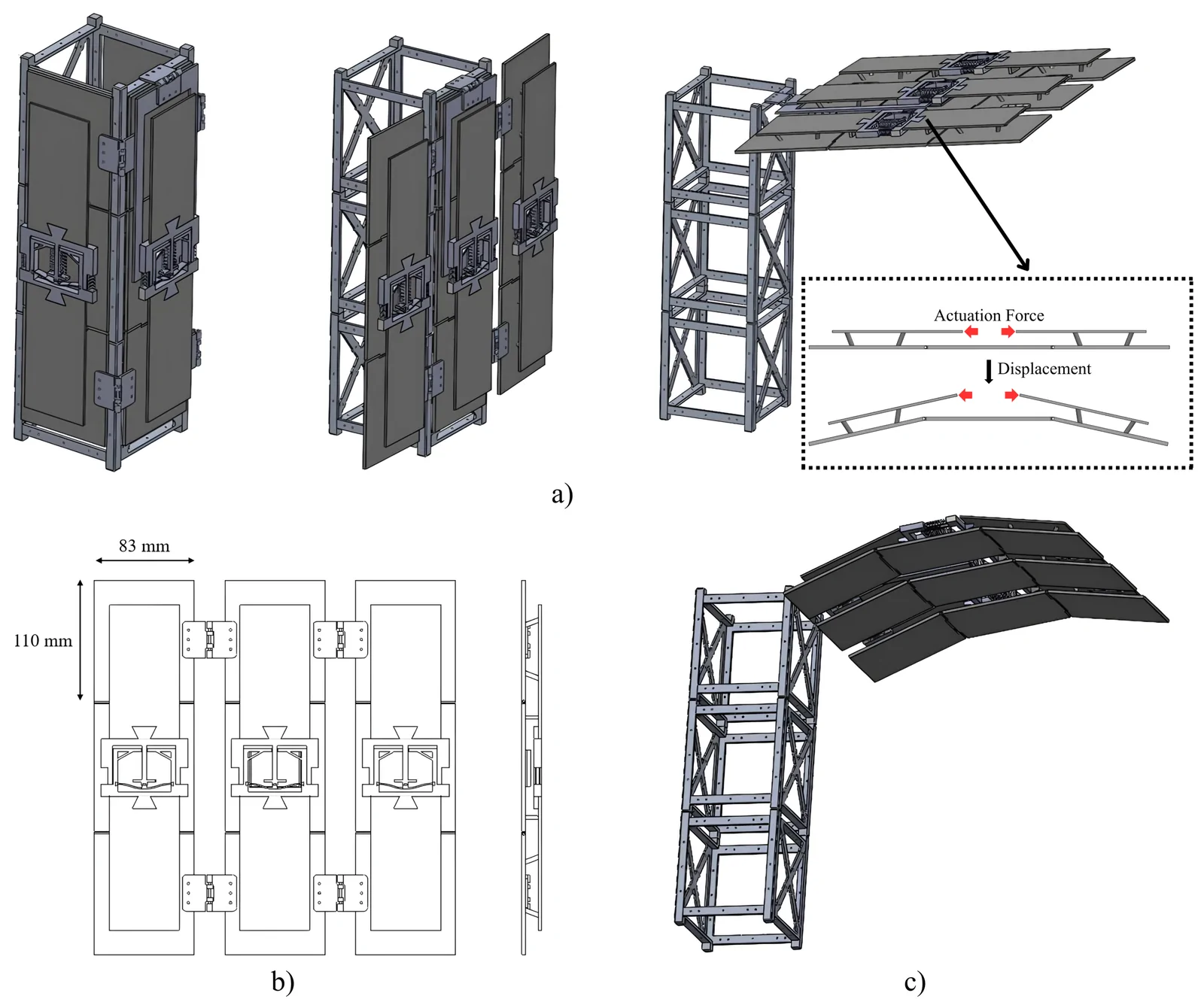
Proposed reflector concept and reconfiguration sequence. (**a**) Stowed, deployed-flat, and actuation-driven reconfiguration stages. (**b**) Single-panel geometry and representative dimensions. (**c**) Final curved reflector configuration. Red arrows indicate actuation forces; black arrows indicate the detail or transition between the illustrated configurations.

The target application is a reflector-panel architecture dimensioned with reference to a 3 U CubeSat chassis. The intended system behavior is binary: a nearly flat configuration for compact deployment-compatible operation and a curved configuration for reflector shaping after deployment. The overall concept adopts nine panels, arranged as three panels per side, with nominal panel dimensions of approximately 83 mm×110 mm. Figure 1a illustrates generic panel deployment on a CubeSat, whereas Figure 1b illustrates panel reconfiguration through articulated joints. These illustrations do not depict specific hardware from the cited literature. References [3,25] provide scientific background for deployment and panel adaptation, respectively. Figure 2a–c presents the proposed reflector concept.

At application-concept level, the intended reflector would be stowed against the CubeSat lateral walls, deployed as a flat array, and subsequently reconfigured into a curved geometry. The present work develops and tests the local switching module and its panel interfaces, rather than experimentally validating this full sequence or the complete reflector. As shown in Figure 2a,c, this concept couples the deployment sequence with panel-level shape change driven by a compact central mechanism. Because continuous SMA powering is poorly compatible with CubeSat power budgets, the actuator was required to minimize holding-power demand and, ideally, consume energy only during state transitions. This motivated the selection of a bistable concept with antagonistic SMA spring pairs, combining reversible switching, passive state retention, and low mass within a compact architecture [29,30].

**Figure 3:**
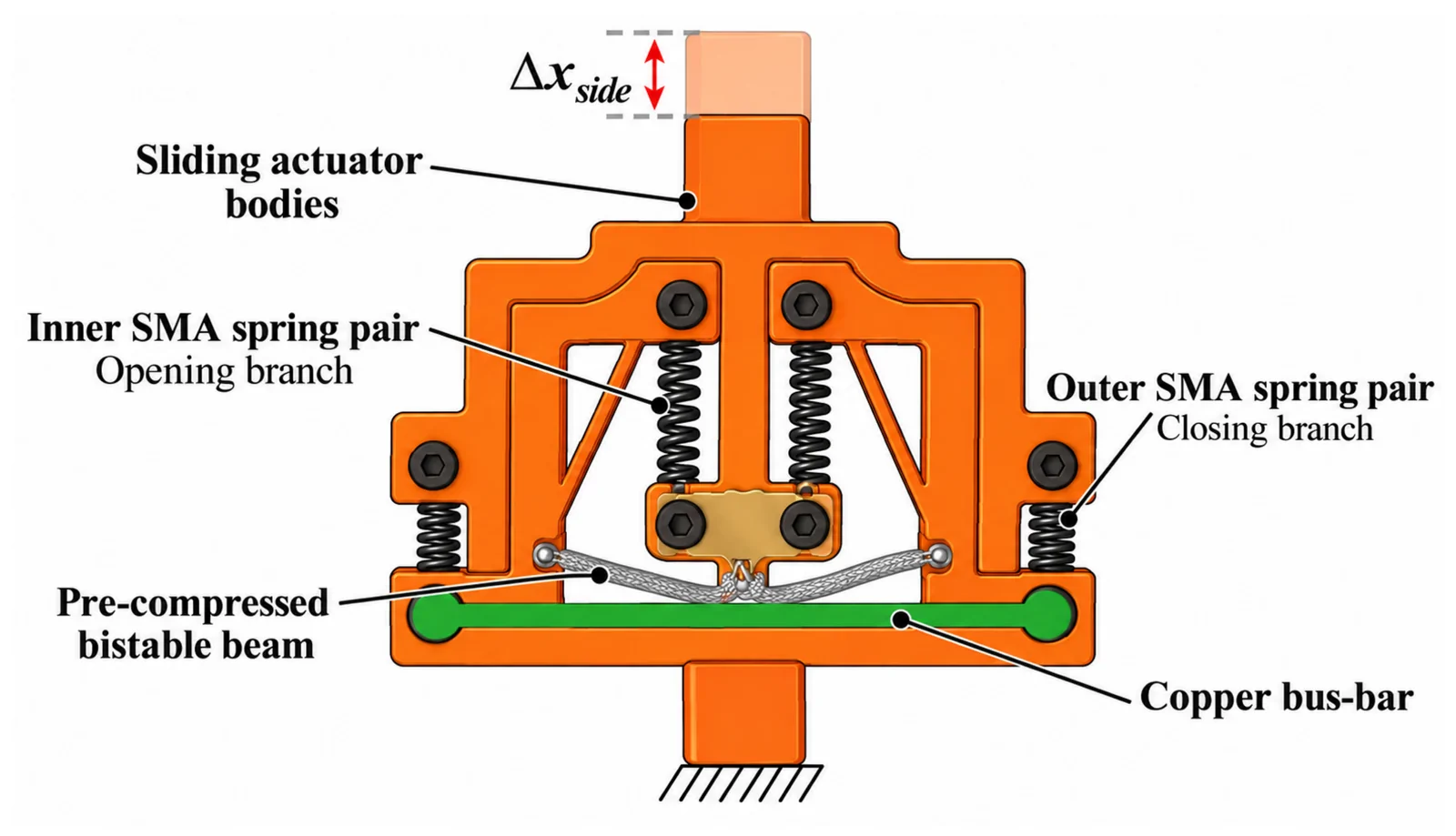
Schematic identification of the actuator components: inner SMA spring pair (opening branch), outer SMA spring pair (closing branch), pre-compressed bistable beam, sliding structural bodies, and copper bus-bar. The red double-headed arrow marks the output-interface displacement. This explanatory illustration is not an experimental photograph. The complete laboratory setup is shown separately in Section 4.1.

To identify the components before introducing the mechanical model, Figure 3 presents a dedicated component-identification schematic based on the actuator view in the experimental setup figure. The inner SMA spring pair drives the opening transition, whereas the outer pair drives the closing transition. The pre-compressed curved beam beneath the central spring attachment provides the bistable core. The sliding structural bodies and the copper bus-bar are also identified; the red double-headed arrow indicates the displacement of the output interface shown. The complete power-supply and measurement setup is presented separately in Section 4.1.

The bistable core retains the switched state without continuous SMA heating. In the panel tests, both SMA channels were set to zero current between pulses while the panel retained its configuration during approximately 60 s of cooling. This establishes zero commanded SMA holding current, not zero consumption of the complete control system. Switching power and a first-order energy estimate are reported in Section 4.1 and Section 4.3.

The reconfiguration module consists of two sliding structural bodies, an internal bistable beam, and two antagonistic SMA spring pairs mechanically arranged in parallel. The inner SMA pair is used to trigger one transition, whereas the outer pair triggers the reverse transition. In this architecture, the SMA elements supply the switching impulse, while the compliant bistable beam defines the stable open and closed states. This division of functions is advantageous because it limits the thermal burden on the active material and reduces the dependence of state retention on sustained heating.

From a development perspective, the design process progressed through staged prototyping: first the bistable beam was assessed numerically, then a bistable-only prototype was built to confirm snap-through behavior, followed by a mechanically complete actuator without active loading, and finally by the fully SMA-driven module and integrated panel demonstrator. This progression reduced development risk and allowed the functional role of each subsystem to be isolated before full integration.

### 2.2. Governing Bistability Relations and Actuator-Sizing Rationale

#### 2.2.1. Bistability Framework

**Figure 4:**
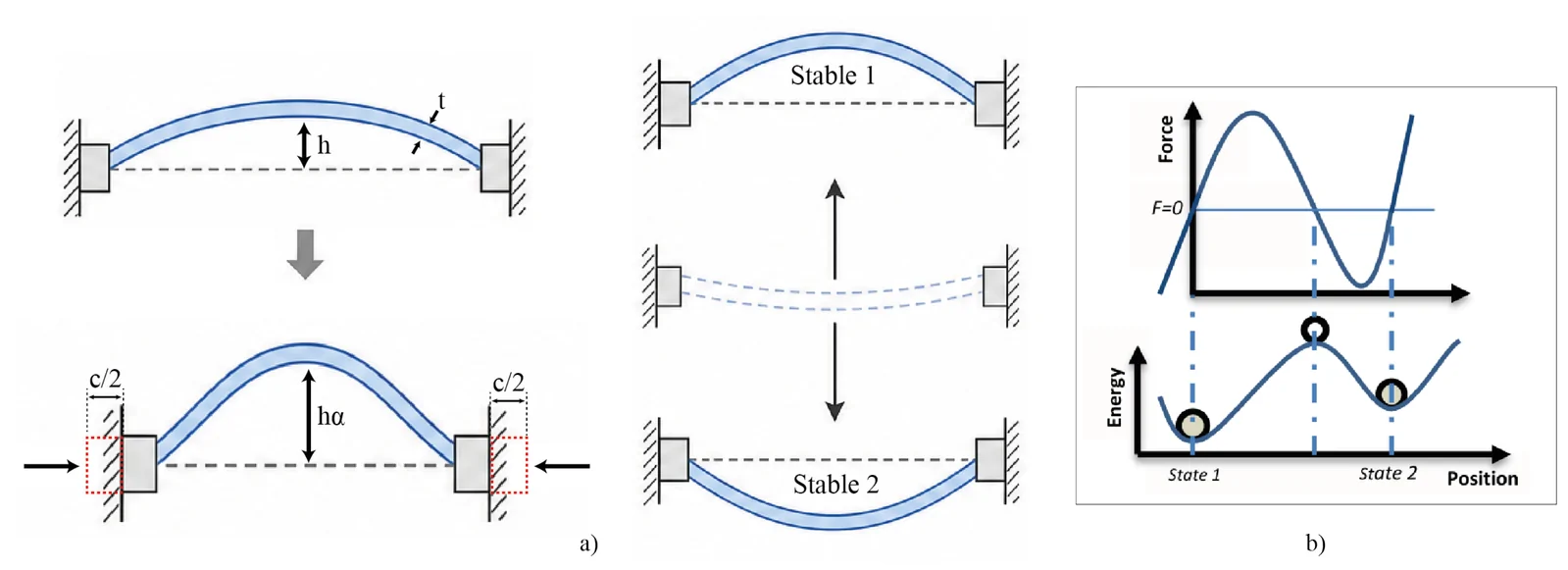
Conceptual schematic of the pre-curved bistable beam. (**a**) Initial geometry, defined by beam rise *h* and thickness *t*, axial pre-compression, and switching between two stable configurations. (**b**) Qualitative force–displacement and potential-energy diagrams illustrating the two stable states. In (**a**), horizontal arrows indicate axial pre-compression and vertical arrows indicate transverse switching; dashed red boxes mark the initial support positions. In (**b**), circled points mark the indicated potential-energy extrema, and dashed guides relate the two diagrams.

Figure 4 summarizes the operating logic of the bistable core in conceptual form. The mechanism starts from a pre-curved compliant beam, then applies axial end shortening to store elastic energy and reshape the force–displacement response, and finally exploits snap-through between two stable configurations. This qualitative schematic is used only to clarify the physical principle; the finite-element model and the numerical results are documented separately through the ABAQUS 2021-based figures discussed below.

**Figure 5:**
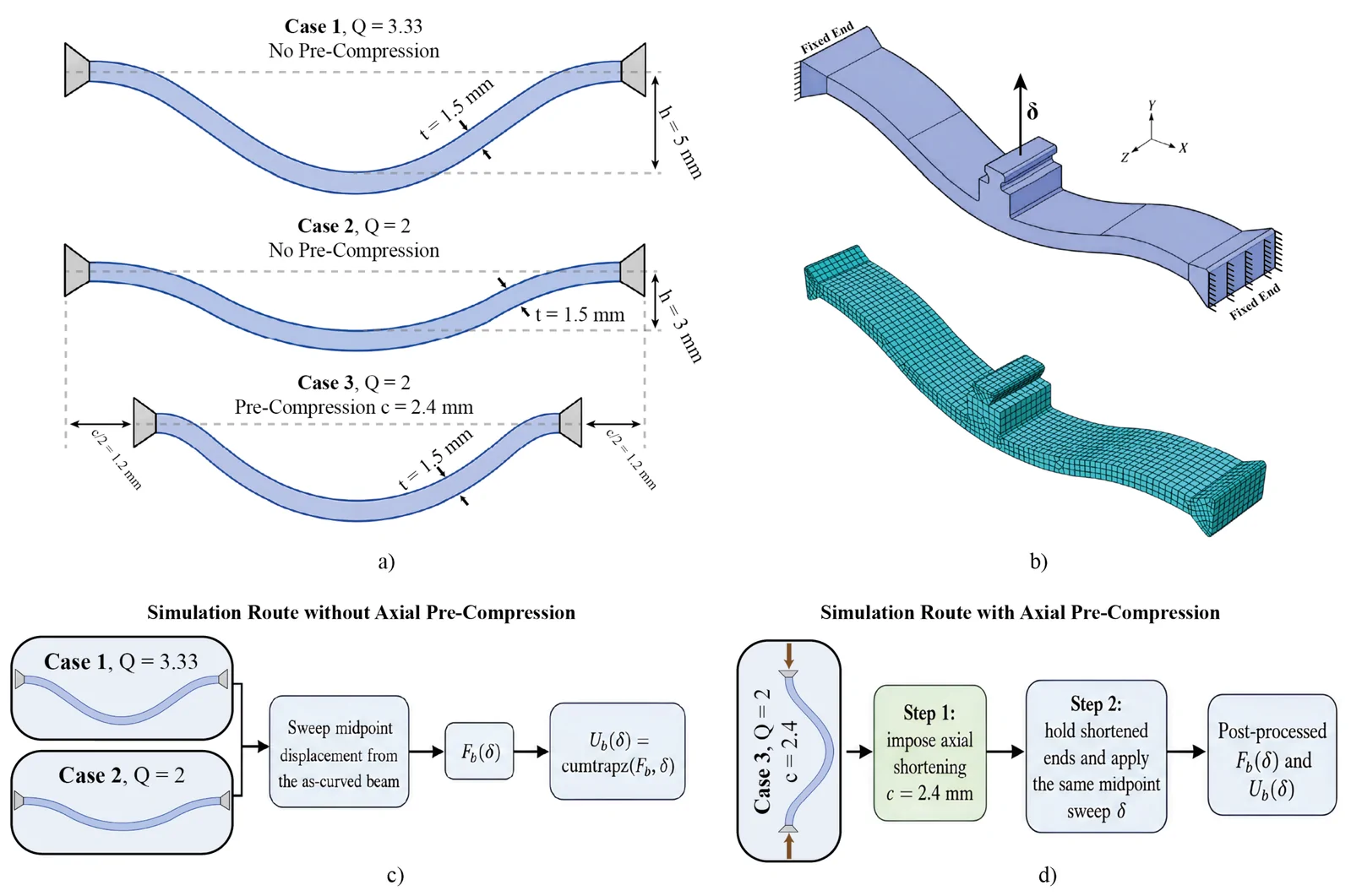
Finite-element workflow for the beam bistability analysis. (**a**) Three beam geometries and their axial pre-compression conditions. (**b**) Common end-constrained model with imposed midpoint displacement and the corresponding mesh. (**c**) Displacement sweep and force–displacement and potential-energy post-processing for Cases 1 and 2. (**d**) Axial pre-compression followed by the midpoint displacement sweep and post-processing for Case 3.

The bistable behavior is generated by a curved compliant beam integrated into the actuator core and subjected to axial pre-compression. The rise-to-thickness ratio Q=h/t is a geometric descriptor, not a measure of curvature. Qiu et al. derived the classical threshold above 2.31 for an initially stress-free, clamped curved beam with the second buckling mode constrained [33]. That result is used here as a reference for the non-pre-compressed cases, not as a universal criterion for the axially shortened TPU beam. In the present design, axial pre-compression is introduced as an additional variable to induce or reinforce bistable behavior even in geometries that would otherwise tend toward monostability. This strategy is consistent with the wider literature on compliant bistable mechanisms for low-energy switching [29,30] and with recent pre-compressed beam-based multistable architectures [34]. Figure 5a summarizes the three beam cases, Figure 5b the common finite-element model, and Figure 5c,d the loading routes adopted for the beam-level analyses.

The present module targets an actuator stroke of approximately 7 mm rather than maximization of travel. The selected 30 mm compliant beam integrates elastic energy storage and snap-through without adding revolute joints or a cam–roller contact to the bistable core. Lever- or cam-based recuperative architectures remain valid alternatives, but adapting them to the same reflector interfaces would require a separate kinematic and packaging study.

The bistable core is described through a small set of geometric and energetic quantities. The primary geometric parameter is the rise-to-thickness ratio

(1) $$Q=\frac{h}{t},$$

where *h* is the initial beam rise and *t* is the beam thickness. Imposed axial end shortening stores compressive elastic energy and introduces a compressive stress state before transverse switching. The geometric stiffness contribution associated with compression changes the balance between stretching and bending energy, and therefore the locations and stability of the equilibria. Under fixed end shortening, this can produce two local energy minima separated by a barrier, even when the uncompressed geometry has only one stable equilibrium. Recovery of bistability is assessed for each pre-compressed case from its computed force and energy paths, rather than inferred from the uncompressed rise-to-thickness threshold alone.

From a mechanics standpoint, the relevant coordinate is the transverse switching displacement δ of the beam midpoint. The corresponding elastic potential associated with the bistable core is written as

(2) $$U_{b}\left(\delta \right)=\int_{0}^{\delta }F_{b}\left(u\right)\;du,$$

where $F_{b}\left(\delta \right)$ is the restoring force obtained numerically. For the discrete force–displacement pairs exported from the finite-element analysis, the potential-energy curve was evaluated in post-processing as

(3) $$U_{b,i}\approx \sum_{k=1}^{i-1}\frac{F_{b,k+1}+F_{b,k}}{2}\left(\delta_{k+1}-\delta_{k}\right),$$

which corresponds to cumulative trapezoidal integration of the numerical data and can be implemented directly in MATLAB R2023a through the cumtrapz routine. In the present context, bistability is identified by the presence of two distinct minima in the resulting potential-energy landscape, separated by an unstable branch of the switching path. For completeness, the total potential during the purely mechanical switching stage can be written as

(4) $$\Pi \left(\delta \right)=U_{b}\left(\delta \right)-W_{\mathrm{ext}}\left(\delta \right),$$

where $W_{\mathrm{ext}}$ is the work of the externally applied switching action. Equilibrium states are then obtained from

(5) $$\frac{d\Pi }{d\delta }=0,$$

whereas local stability requires

(6) $$\frac{d^{2}\Pi }{d\delta^{2}}>0.$$

Bistability is therefore associated with two distinct minima of Π(δ) separated by an unstable branch. In practical terms, the snap-through event is reached at a limit configuration for which the net generalized force vanishes, and the tangent stiffness changes sign,

(7) $$F_{\mathrm{net}}\left(\delta \right)=0,\;\frac{dF_{\mathrm{net}}}{d\delta }=0.$$

To support this framework, a dedicated beam-level numerical campaign was carried out in ABAQUS 2021 on the pre-curved and pre-compressed compliant beam. The beam was modeled as an elastic TPU component with a Young’s modulus of approximately 39 MPa and a Poisson ratio of 0.24. This material choice is also consistent with recent pre-compressed multistable beam studies based on thermoplastic polyurethane-like elastomeric behavior [34]. The analyses were performed as geometrically nonlinear static simulations with end-constrained boundary conditions and an imposed midpoint transverse displacement, so that the restoring-force history and the corresponding energy evolution could be extracted. Quadratic brick elements (C3D20) with an average mesh size close to 0.6 were adopted. Figure 5b shows the common ABAQUS setup, whereas Figure 5c,d report the two loading routes considered in the study, namely the direct midpoint sweep used for the non-pre-compressed cases and the two-step pre-loading-plus-sweep sequence used to obtain the pre-compressed response.

The beam analyses were used to screen geometries and estimate switching stroke and reaction-force scale for preliminary actuator sizing. No mesh-refinement study or independent measured force–displacement curve of the isolated beam is reported, so discretization error and quantitative beam-model accuracy have not been established. The numerical force and energy paths are therefore design-support estimates, not a validated predictive model transferable to other geometries, materials, or loading conditions. The assembled-actuator switching tests provide functional evidence for the selected mechanism, but do not independently validate the computed beam force curve or energy barrier.

**Figure 6:**
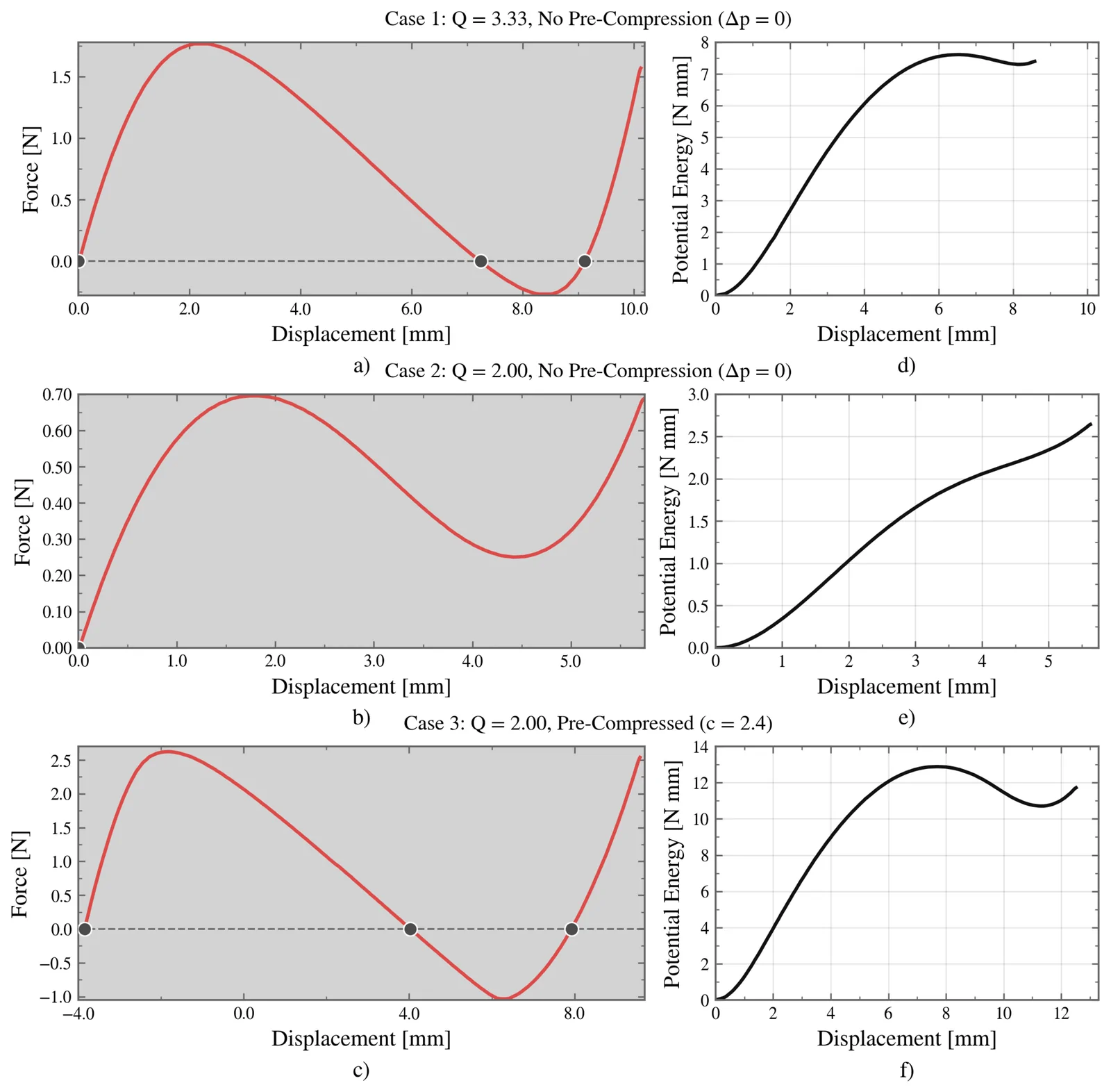
Numerical signatures of bistable and monostable beam behavior. (**a**,**d**) Case 1 (Q=3.33, no pre-compression), showing bistability. (**b**,**e**) Case 2 (Q=2, no pre-compression), showing monostable behavior. (**c**,**f**) Case 3 (Q=2, with approximately 2.4 mm axial pre-compression), showing recovery of bistability. Red curves are the restoring-force responses, black curves are the corresponding potential-energy profiles, horizontal dashed lines indicate zero force, and circular markers identify the indicated zero-force points.

Three representative cases were then investigated to establish the bistability logic and to guide miniaturization. In the first case, the beam geometry was defined by h=5 mm and t=1.5 mm, corresponding to Q=3.33. The numerical force–displacement response displayed the expected bistable trend, and the associated potential-energy profile exhibited two local minima, showing bistability in this simulated case, consistent with the classical constrained-beam reference. This comparison does not establish the applicability or accuracy of that threshold for the present TPU model. In the second case, the rise was reduced to 3 mm while the thickness was kept at 1.5 mm, so that Q=2. Under the same loading concept, the response became monostable, with loss of the second stable branch and collapse to a single energy well. In the third case, the same Q=2 geometry was combined with an axial pre-compression of approximately 2.4 mm. Under these conditions, the beam recovered a bistable response, with two stable configurations reappearing in both the force-based and energy-based interpretations. These three cases are collected in Figure 6a–f and Table 1.

**Figure 7:**
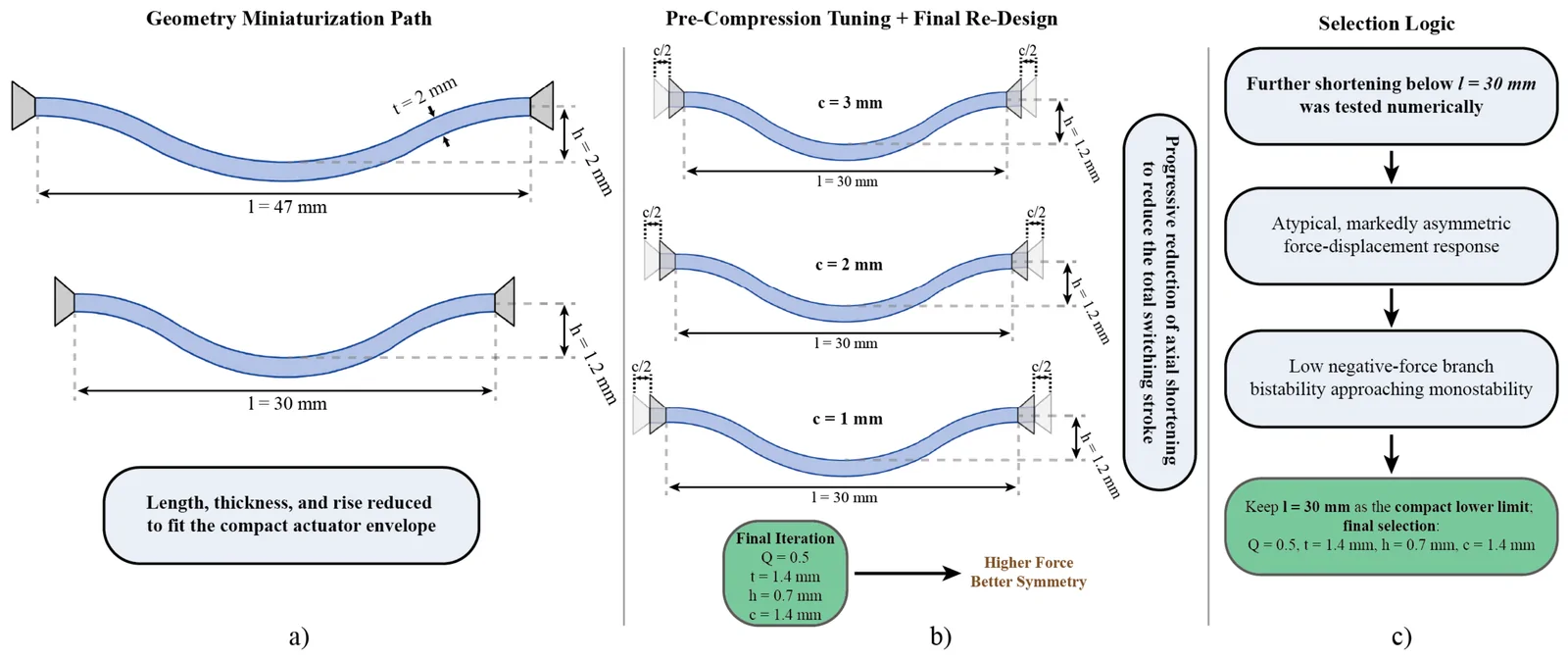
Beam miniaturization and final selection logic. (**a**) Geometry miniaturization from the early 47 mm-long concept to the compact 30 mm beam, with simultaneous reduction of thickness and rise. (**b**) Pre-compression tuning of the compact beam from c=3 mm to c=1 mm to reduce switching stroke, followed by the final redesign step with Q=0.5, t=1.4 mm, h=0.7 mm, and c=1.4 mm to recover force and symmetry. (**c**) Selection logic showing why beam lengths below 30 mm were rejected and why the final compact configuration was retained.

**Figure 8:**
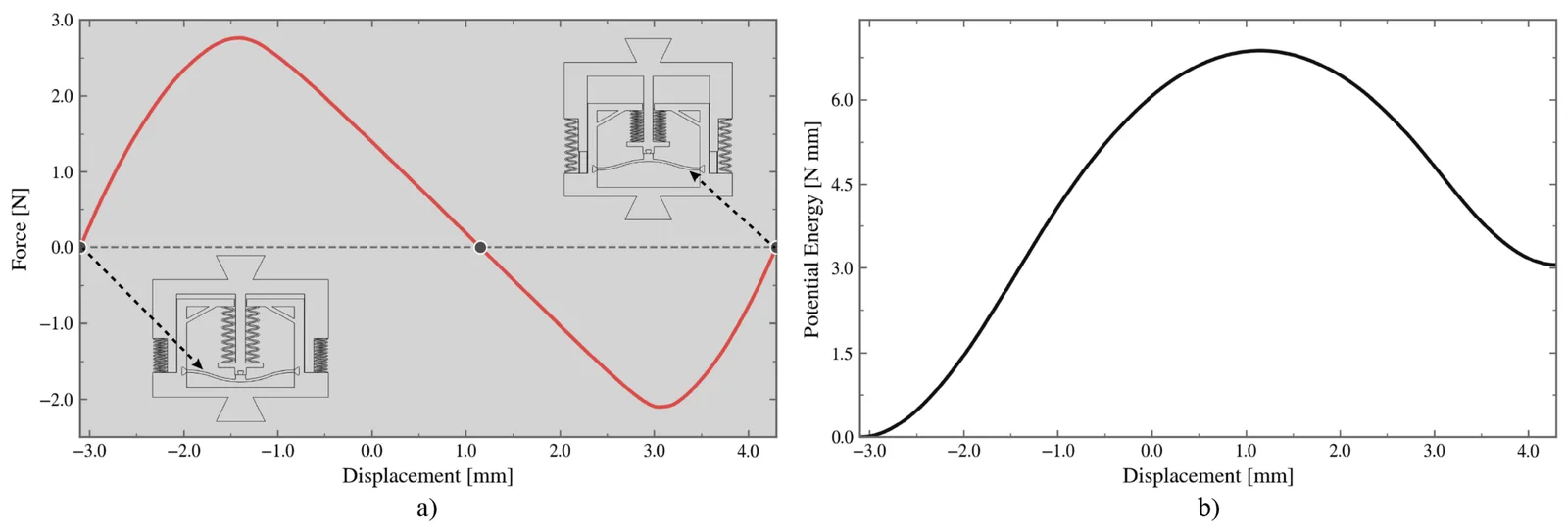
Final bistable response of the selected compact beam configuration (Q=0.5, t=1.4 mm, h=0.7 mm, c=1.4 mm). (**a**) Numerical force–displacement curve of the final switching path, annotated with the two stable configurations of the actuator core. (**b**) Corresponding potential-energy profile obtained by integrating the numerical restoring-force response. The red curve in (**a**) is the restoring-force response; the horizontal dashed line indicates zero force, circular markers identify the indicated equilibrium points, and dashed arrows connect the end configurations to the actuator-core sketches. The black curve in (**b**) is the corresponding potential energy.

Once the bistability logic had been established, the numerical campaign moved to beam miniaturization. An early beam concept occupied about 47 mm of length and relied on approximately 3 mm of pre-compression, as summarized in Figure 7a. Subsequent iterations reduced the beam length to 30 mm, while the thickness and initial rise were both reduced from 2 mm to 1.2 mm, again as shown in Figure 7a. Additional iterations then tuned the pre-compression from 3 mm to 2 mm and finally to 1 mm, with the explicit goal of reducing the total transverse stroke to a value compatible with the compact actuator architecture, as illustrated in Figure 7b. Further attempts to reduce the beam length below 30 mm were also explored during the simulation campaign, but they produced atypical and markedly asymmetric force–displacement curves, together with low negative-force levels indicative of bistability approaching monostability. For this reason, a beam length of 30 mm was retained as the practical lower limit for the present design, consistent with the selection logic reported in Figure 7c. At that stage, however, the lowest-stroke compact case still showed insufficient force and poor symmetry. A further iteration was therefore introduced by reducing *Q* and increasing axial pre-compression, since pre-compression had proved to affect the reaction force much more strongly than pre-curvature alone, as highlighted in Figure 7b. The final selected geometry adopted t=1.4 mm and h=0.7 mm, corresponding to Q=0.5, together with an axial pre-compression of c=1.4 mm. The full miniaturization and selection logic is summarized in Figure 7, while the corresponding final force–displacement and potential-energy response is reported in Figure 8a,b. This final compromise raised the computed peak switching force to about 2.8 N from about 1.7 N and yielded a maximum beam transverse displacement of approximately 7.4 mm. This selected configuration provided the mechanical basis for the final SMA-driven actuator.

#### 2.2.2. SMA Spring Sizing Rationale

Helical SMA springs were selected to provide the required switching stroke within the compact axial space available, whereas a directly coupled straight-wire solution would require a longer active path or a displacement-amplification mechanism. The SMA elements trigger the transitions, while the bistable core provides passive state retention; the selection therefore prioritizes packaging and switching capability rather than maximum active-element stiffness. Straight SMA wires remain a valid alternative, but no matched comparison of actuation current or energy was performed.

**Figure 9:**
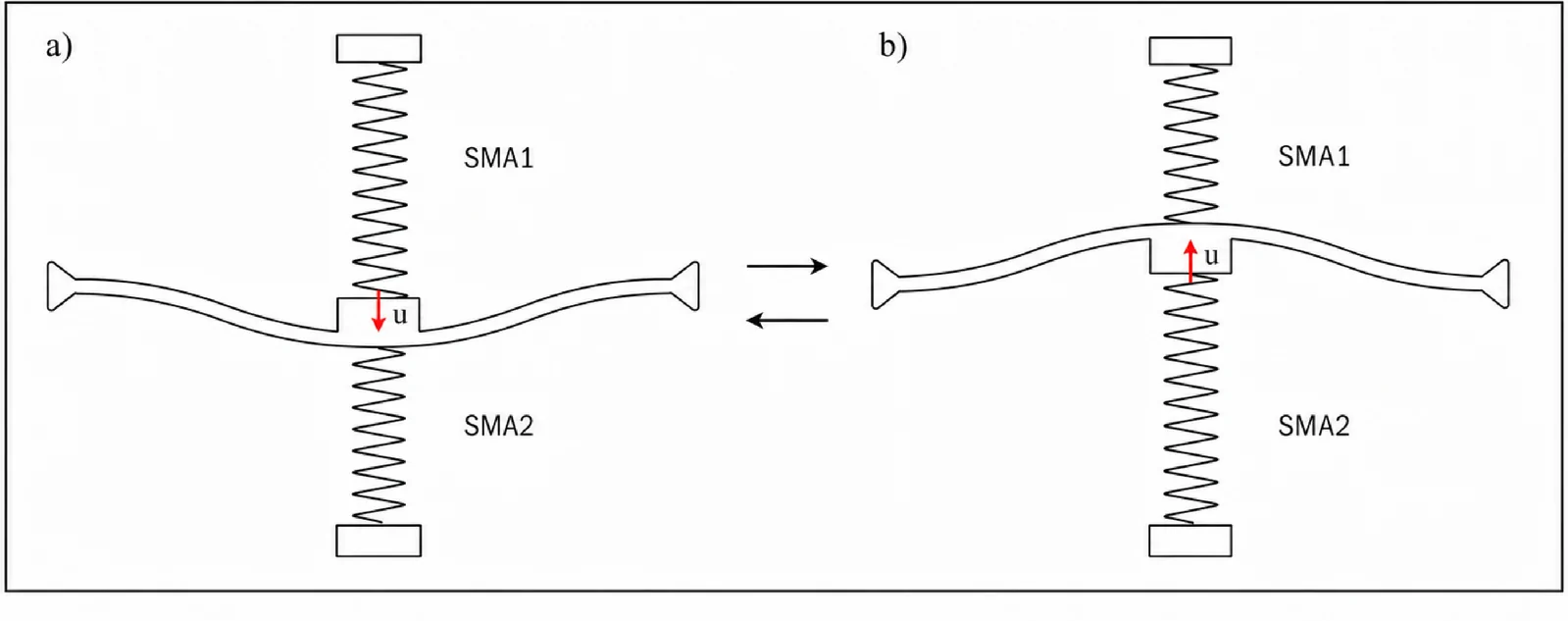
Spring-selection logic of the bistable beam–SMA system. (**a**) Lower stable configuration with downward displacement *u*. (**b**) Upper stable configuration after snap-through, with upward displacement *u*. Each SMA branch is drawn as a single equivalent spring, representing the two real SMA springs mounted in parallel in the prototype.

The spring-sizing logic is summarized in Figure 9a,b, which shows the two stable configurations of the bistable beam and the two antagonistic SMA branches acting on the central shuttle. For clarity, each branch is represented by a single equivalent spring, although the physical implementation uses two identical SMA springs in parallel per branch.

During opening, the inner branch is the active driving branch and the opposite branch acts as the passive antagonist. The net switching force can therefore be written as

(8) $$F_{\mathrm{net}}^{\mathrm{open}}\left(\delta \right)=F_{\mathrm{SMA},\mathrm{in}}^{A}\left(\delta \right)-F_{b}\left(\delta \right)-F_{\mathrm{SMA},\mathrm{out}}^{M}\left(\delta \right)-F_{f}\left(\delta \right),$$

where $F_{\mathrm{SMA},\mathrm{in}}^{A}$ is the force delivered by the activated SMA pair on the branch that pulls the shuttle toward the target state, $F_{b}$ is the restoring reaction of the bistable beam, $F_{\mathrm{SMA},\mathrm{out}}^{M}$ is the passive resisting force of the opposite SMA pair, and $F_{f}$ collects frictional losses in the transmission. Friction is retained in the general force balance. The numerical screening below used a zero-friction reference case for preliminary spring selection; this modeling simplification is not experimental evidence that friction is negligible in the assembled prototype. Here, δ denotes the shuttle displacement along the switching path. The symmetric closing condition becomes

(9) $$F_{\mathrm{net}}^{\mathrm{close}}\left(\delta \right)=F_{\mathrm{SMA},\mathrm{out}}^{A}\left(\delta \right)-F_{b}\left(\delta \right)-F_{\mathrm{SMA},\mathrm{in}}^{M}\left(\delta \right)-F_{f}\left(\delta \right).$$

In this second case, the outer branch becomes the active one, while the inner branch provides the passive opposing contribution. The superscripts *A* and *M* denote the active austenitic and passive martensitic conditions, respectively.

For a successful state transition, the active spring pair must drive the mechanism through the unstable region, so that the sufficient sizing inequalities are

(10) $$F_{\mathrm{SMA},\mathrm{in}}^{A}>F_{b}\left(\delta \right)+F_{\mathrm{SMA},\mathrm{out}}^{M}+F_{f},$$

(11) $$F_{\mathrm{SMA},\mathrm{out}}^{A}>F_{b}\left(\delta \right)+F_{\mathrm{SMA},\mathrm{in}}^{M}+F_{f}.$$

These relations are not intended as a full constitutive SMA law; rather, they formalize the design requirement visible in Figure 9a,b: in either switching direction, the active branch must overcome the beam reaction, the passive antagonist branch, and any friction contribution. A zero-friction screening case provides a nominal force budget, whereas verification of the assembled mechanism requires the friction contribution along the switching path.

The SMA spring was selected through a compactness-first screening combined with a first-order force verification. The available space between the spring fixing points was approximately 6 mm. For a helical spring in a compact configuration, the corresponding solid length can be approximated as

(12) $$L_{b}=d\left(n+1\right),$$

where *d* is the wire diameter and *n* is the number of active coils. The selected laboratory spring had a wire diameter d=0.5 mm and outer diameter OD=3 mm, hence a mean diameter D=OD−d=2.5 mm. Although the nominal 6 mm space would ideally allow about eleven active coils, fixation constraints reduced the effective working length to about 4 mm. A conservative value of n=7 active coils was therefore adopted.

In the initial assembled configuration, one antagonistic spring pair is axially pre-extended by approximately 40 percent relative to the compact length of the opposite pair, with both pairs having the same number of active turns.

The NiTi springs were purchased from Xian Zhanwo Metal Material Co., Ltd. (Xi’an, China) No additional heat treatment was applied by the authors. Before installation, the springs underwent 20 consecutive heating–cooling actuation cycles under a constant axial load close to the operating load, as thermomechanical preconditioning intended to reduce initial response transients. This preparation follows the conditioning approach used in the authors’ previous NiTi-wire study [35], but is distinct from the subsequent ten-cycle test of the assembled module and does not establish long-term fatigue resistance. Their material properties were experimentally identified in the authors’ previous characterization campaigns [36,37]. The wire and outer diameters correspond to Type 2 in Reference [37], whose Tables 1 and 2 identify the spring type and its phase-dependent shear moduli. The published mechanical, thermal, and electrically driven recovery data are referenced rather than reproduced here; the following linear relations are first-order sizing estimates, not measured force–displacement–temperature curves of the assembled bistable module.

Once the spring geometry had been fixed, the spring constant was estimated from the classical expression for a round-wire helical spring,

(13) $$K=\frac{Gd^{4}}{8D^{3}n},$$

where *G* is the shear modulus of the material. Using the experimentally identified phase-dependent shear moduli for Type 2 reported in Table 2 of Reference [37], namely $G_{A}\approx 31,000$ MPa and $G_{M}\approx 15,000$ MPa, gives

(14) $$K_{A}=\frac{G_{A}d^{4}}{8D^{3}n}=\frac{31,000\;{\left(0.5\right)}^{4}}{8\;{\left(2.5\right)}^{3}\;7}\approx 2\;N/\mathrm{mm},$$

(15) $$K_{M}=\frac{G_{M}d^{4}}{8D^{3}n}=\frac{15,000\;{\left(0.5\right)}^{4}}{8\;{\left(2.5\right)}^{3}\;7}\approx 1\;N/\mathrm{mm}.$$

Assuming as a first-order estimate that the useful actuator stroke is about 7 mm, Hooke’s law gives the force of a single spring as

(16) $$F_{A}=K_{A}\;\Delta \approx 2\;N/\mathrm{mm}\times 7\;\mathrm{mm}\approx 14\;N,$$

(17) $$F_{M}=K_{M}\;\Delta \approx 1\;N/\mathrm{mm}\times 7\;\mathrm{mm}\approx 7\;N.$$

Since each actuator branch uses two identical springs in mechanical parallel, the force available from one active SMA pair is approximately 28 N, whereas the opposing passive pair contributes about 14 N. The beam-level numerical campaign had already shown that the peak force required to drive snap-through was about 3 N. Retaining transmission friction, the nominal sizing condition is

(18) $$F_{\mathrm{SMA},1,2}^{A}>F_{\mathrm{SMA},1,2}^{M}+F_{b}^{\max}+F_{f}\left(\delta \right),\;28\;N>14\;N+3\;N+F_{f}\left(\delta \right),$$

The quoted nominal forces leave a residual budget of 11 N for friction and other unmodeled resisting loads. This is a conditional sizing budget, not a measured friction bound or a verified minimum margin over the 7 mm stroke: the active and passive SMA forces vary with displacement and thermal state. Hinge, guide, and transmission losses were not measured separately, and the observed switching does not establish their individual contributions. This procedure should be interpreted as preliminary spring selection rather than a full-path force-margin validation or a full multiphysics SMA optimization.

In the panel-interface kinematic model, the actuator stroke is transferred symmetrically to the two mirrored panel-driving interfaces, so that the kinematic input used in the panel model can be written as

(19) $$\Delta x_{\mathrm{side}}\approx \frac{\Delta x_{\mathrm{act}}}{2}.$$

With the experimentally targeted actuator stroke close to 7 mm, this relation leads to an imposed side displacement of approximately 3.5 mm per interface in the reflector simulation.

The prototype geometry, material-model assumptions, and actuation settings are summarized in Table 2.

## 3. Prototype Development, Numerical Refinement, and Panel-Interface Integration

### 3.1. Module Assembly and Application-Concept Constraints

The integrated demonstrator was developed progressively, starting from the compliant bistable beam and then transferring the verified beam logic into a compact actuator composed of two sliding bodies, an internal pre-compressed beam, and the interfaces required for two antagonistic SMA spring pairs. The actuator was finally integrated with mirrored reflector-panel interfaces to convert the local stroke into panel rotation and array-level curvature change. The packaging reference was a nine-panel reflector concept organized as three identical arrays of three sandwich panels within the 3U CubeSat envelope, with panel thickness limited to about 16 mm. This system layout defines the intended integration constraints; the experiments reported here concern the actuator and integrated panel demonstrator, not the assembled nine-panel reflector. Adjacent panels were connected through lower hinges with nominal 2 mm metal pins, while the upper region hosted the actuator interface. The actuator therefore had to remain compact while still providing an effective stroke of about 7 mm, and these packaging constraints directly shaped the beam miniaturization strategy, the spring geometry, and the actuator-housing layout.

### 3.2. Motion Analysis and Kinematic Transfer

The actuator-to-panel kinematic transfer was defined through a SolidWorks 2025 motion study on one representative array, since the three lateral arrays were geometrically identical. Using the actuator stroke as input, the kinematic model imposed a symmetric side displacement of about 3.5 mm per interface for a total actuator stroke of about 7 mm, i.e.,

(20) $$\Delta x_{\mathrm{side}}\approx \frac{\Delta x_{\mathrm{act}}}{2}.$$

**Figure 10:**
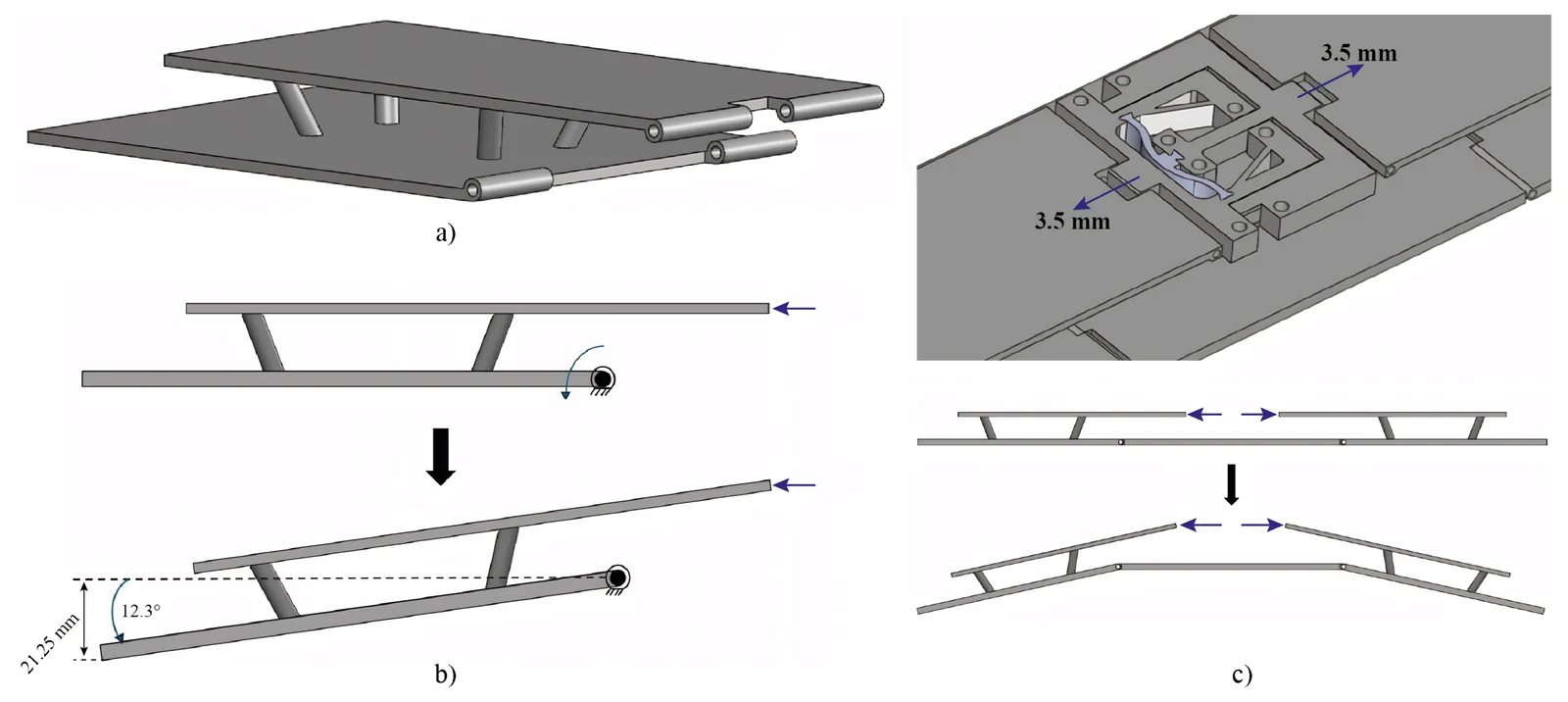
Panel kinematic model used to transfer the actuator stroke to reflector reconfiguration. (**a**) Detail of the hinged panel connection. (**b**) Side-view kinematics of a panel rotating about the lower hinge. (**c**) Symmetric actuator-to-panel displacement transfer and the resulting array-level shape change. Blue arrows indicate imposed displacement directions, curved arrows indicate panel rotation, and black arrows indicate transitions between the illustrated configurations. The angles and displacements shown are simulation outputs, not measured surface metrics.

The two mirrored interfaces were driven by displacement-controlled linear motors acting on the upper contact faces of the outer panels and reaching the target displacement in about 5 s. The simulation predicted a cylindrical-like reconfigured shape with a maximum panel rotation of about $12.3^{{}^{\circ}}$ and a maximum transverse edge displacement of about 21.3 mm. The 12.3-degree rotation is a kinematic prediction only, not an experimentally measured panel angle. It also exposed the main weakness of the initial dovetail-type actuator–panel coupling, namely interference and excessive friction during rotation. This directly motivated the transition to a hinge-based coupling, implemented by modifying the actuator terminals and adopting the same nominal 2 mm pinned logic already used between adjacent panels. Figure 10 summarizes the panel-level kinematic transfer and the resulting reflector response.

### 3.3. Numerically Guided Structural Refinement

The PETG actuator bodies were manufactured for laboratory proof-of-concept testing, not selected as a flight-ready or high-temperature housing material. As a commercial reference, the PETG Basic datasheet from Bambu Lab (Shenzhen, China) reports a glass-transition temperature of 69 degrees Celsius and heat-deflection temperatures of 68 degrees Celsius at 1.8 MPa and 71 degrees Celsius at 0.45 MPa. These standardized specimen values are not allowable operating temperatures of the assembled actuator; they highlight the need to distinguish the measured SMA temperature from the unmeasured local temperature of the load-bearing polymer. The structural analyses below do not establish elevated-temperature strength or creep resistance.

**Figure 11:**
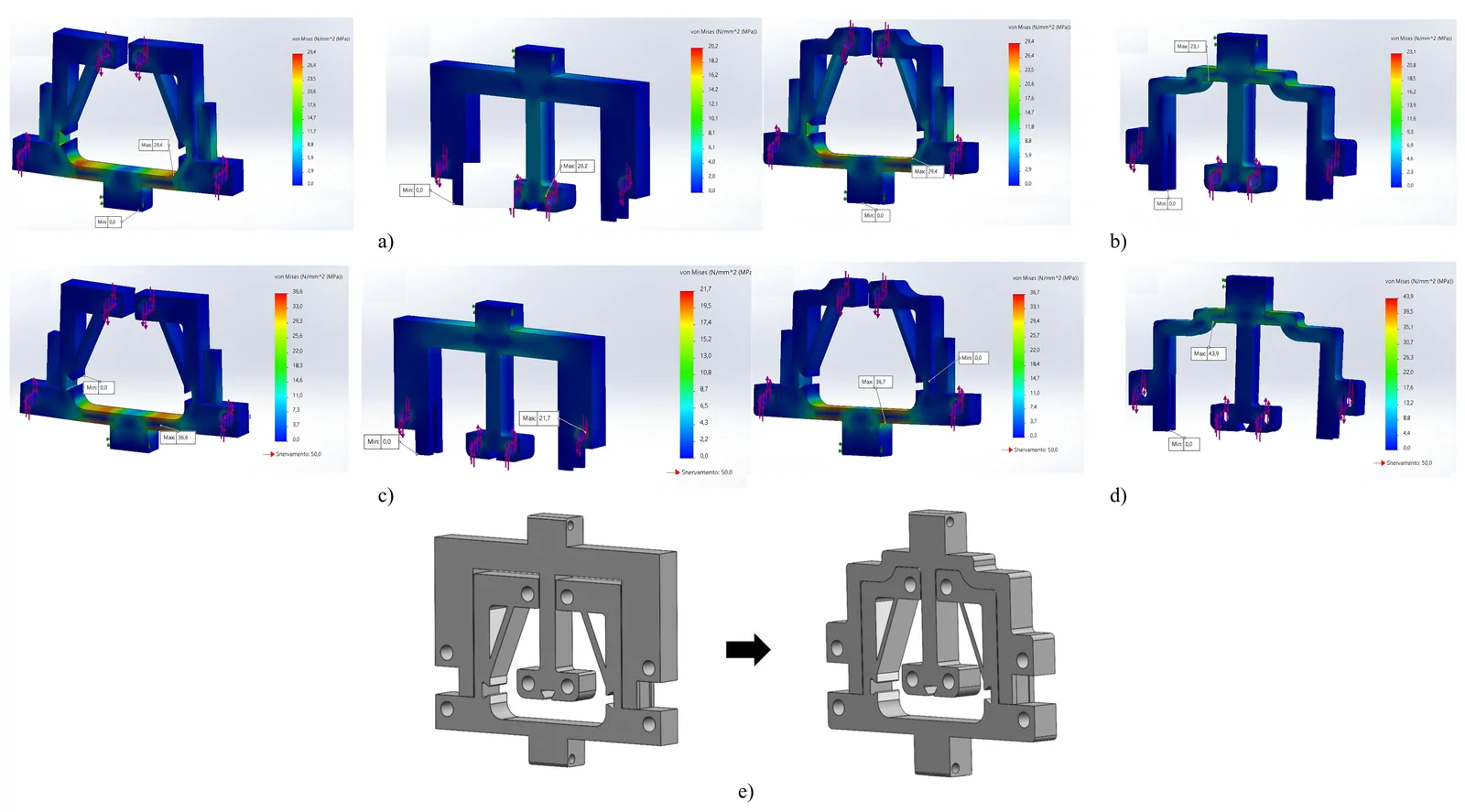
Numerical optimization of the actuator bodies. (**a**,**b**) Von Mises stress maps for the two actuator bodies under the first switching load case. (**c**,**d**) Corresponding stress maps under the opposite switching load case. (**e**) Transition from the initial actuator-body geometry to the optimized lightweight configuration.

After the motion-transfer path had been defined, finite-element analyses in SolidWorks 2025 were used to refine both the actuator and the reflector panels. The two actuator bodies were analyzed separately, with fixed constraints at the hinge locations and concentrated loads at the spring-attachment points. Two switching load cases were considered to represent the opposite operating directions: in the opening-oriented case, the inner SMA pair provided about 14 N while the outer pair contributed about 7 N, whereas in the closing-oriented case the force distribution was reversed. The corresponding stress maps for the two bodies and the two load cases are reported in Figure 11a–d.

The initial actuator geometry contained low-utilization regions under the analyzed load cases, according to the PETG yield reference adopted for the assessment. These results motivated geometric lightweighting of those regions while preserving the main transmission paths, hinge regions, and SMA-interface details; they do not establish a factor of safety above 2 throughout the bodies.

As summarized in Figure 11e, the actuator-body mass was reduced from about 13 g to about 10 g, corresponding to approximately 23%. The optimized geometry was then rechecked under both switching cases. The most demanding condition was the closing-oriented one, where the upper body reached a localized minimum factor of safety of about 1.14. This is the minimum factor obtained for the analyzed prototype load case, not a qualified structural margin. The SMA forces were estimated using a preliminary linearized screening approach rather than a validated full-path load characterization.

A similar refinement strategy was applied to the reflector panels, with the dual objective of reducing mass while maintaining local stiffness near the hinge and actuator-interface regions. Material was removed from broad low-stress regions while stiffness and geometric reliability were preserved where load transfer occurred. Excessive compliance in the central panel area was corrected by adding small central supports, and the hinge geometry was revised to accommodate the nominal metal pins without weakening the panel extremities. The final solution reduced the nominal panel thickness from about 3 mm to about 2 mm while locally reinforcing the hinge regions.

This redesign improved both mass efficiency and expected structural robustness. At the component level, the mass of each lateral panel decreased from about 38.12 g for the initial PLA version to about 31.27 g for the optimized Nylon-Carbon version, whereas the central panel decreased from about 20.01 g to about 12.60 g. For the assembly assessed in this refinement, these changes reduced the reported mass from about 112.25 g to 88.76 g, i.e., by about 20.93%. Overall, the refinement stage improved actuator compactness, reflector kinematics, and structural robustness simultaneously.

## 4. Results and Discussion

### 4.1. Experimental Setup and Test Protocols

The experimental campaign was organized to validate first the stand-alone actuator and then the integrated reflector-panel demonstrator. The instrumentation included a programmable power supply, a Micro-Epsilon laser displacement sensor (Ortenburg, Germany), an HBM QuantumX acquisition unit (HBM, Darmstadt, Germany), and a FLIR A615 infrared camera (FLIR Systems AB, Täby, Sweden), so that the electrical input, mechanical output, and thermal response of the SMA-driven system could be observed simultaneously.

Non-contact temperature observations used a FLIR A615 infrared camera, with 640 by 480 pixels and a manufacturer-specified thermal sensitivity (NETD) below 0.05 degrees Celsius at 30 degrees Celsius. The nominal temperature accuracy is plus or minus 2 degrees Celsius or 2 percent of the reading [38]; NETD is not absolute accuracy or measurement uncertainty. The authors’ earlier studies describe A615-based NiTi characterization [35,39]; the Kapton-assisted method in the latter used an emissivity of 0.97, whereas no Kapton was applied to the springs in the present tests.

For bare NiTi, da Silva et al. reported emissivities of approximately 0.171 in martensite and 0.155 in austenite during heating, using an 8–14 micrometer spectral band [40]. An emissivity of approximately 0.17 is therefore a literature reference, not a verified record of the camera setting used here or a specimen-specific calibration. No independent temperature verification was performed in this campaign. Surface condition, phase-dependent emissivity, reflected radiation, and finite spatial resolution introduce contributions not quantified here; the manufacturer accuracy cannot be treated as the complete uncertainty of these spring-temperature measurements. A combined uncertainty budget for temperature and displacement is not available, so the reported values are approximate proof-of-concept observations.

**Figure 12:**
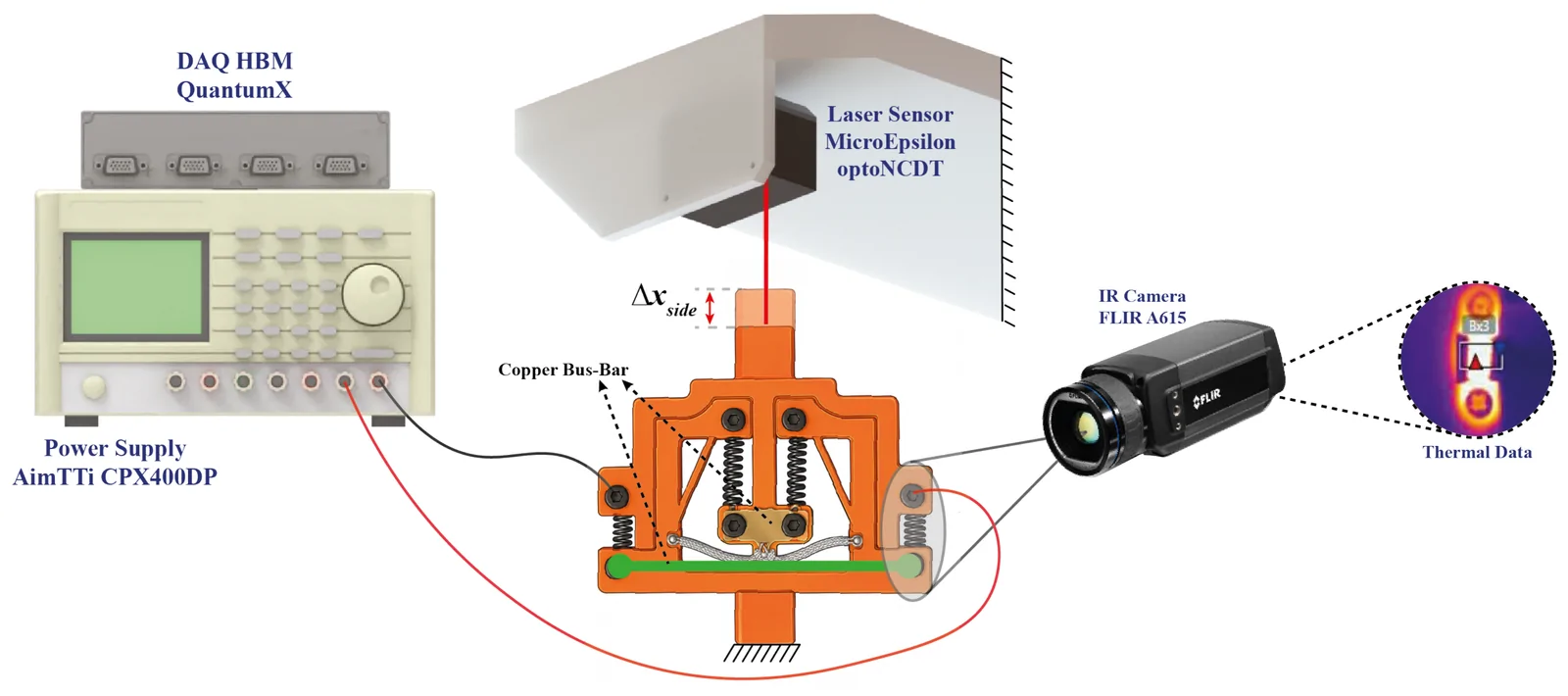
Experimental setup and measurement chain used for actuator validation. The figure identifies the programmable power supply, the HBM QuantumX acquisition unit, the Micro-Epsilon laser displacement sensor, the FLIR thermal camera, the actuator specimen, and a representative thermal frame acquired during testing.

The actuator components are identified in Figure 3, whereas Figure 12 shows the complete laboratory setup and measurement chain.

At the actuator level, two current ramps were applied over a total test duration of approximately 130 s. Each pulse reached a peak current close to 3 A after about 10 s. The first pulse activated the internal SMA spring pair and drove the module toward the second stable state, whereas the second pulse, delayed by roughly 100 s, activated the outer SMA pair and returned the mechanism to the initial configuration.

For the integrated-panel protocol, each activated branch comprised two SMA springs connected electrically in series, with approximately 1.5 V across the pair at 3 A, corresponding to a peak branch power of 4.5 W. The current rose approximately linearly from zero to 3 A in 3 s and was switched off immediately at the peak, with no holding plateau. Only one branch was energized at a time, and both channels were set to zero current between pulses.

At panel level, the same actuation principle was retained with faster ramps. In this case, both control channels reached 3 A in approximately 3 s, and the delay between the two channels was about 61 s. Under these conditions, the panel demonstrator switched between nearly flat and curved mechanical configurations; these tests did not characterize complete-reflector surface accuracy or electromagnetic performance.

During the approximately 60 s interval between transitions, the springs cooled toward the ambient baseline while the panel retained its curved configuration at approximately 19 mm edge displacement. This is an observed approximate cooldown under the laboratory protocol, not a measured minimum cooling time.

A repeatability protocol involving ten complete opening–closing cycles was then carried out over approximately 1340 s. The purpose of this sequence was not to establish durability or qualification limits, but to verify whether the integrated module could preserve repeatable short-term switching behavior under the investigated laboratory conditions.

### 4.2. Experimental Validation of the Actuator and Integrated Panel

**Figure 13:**
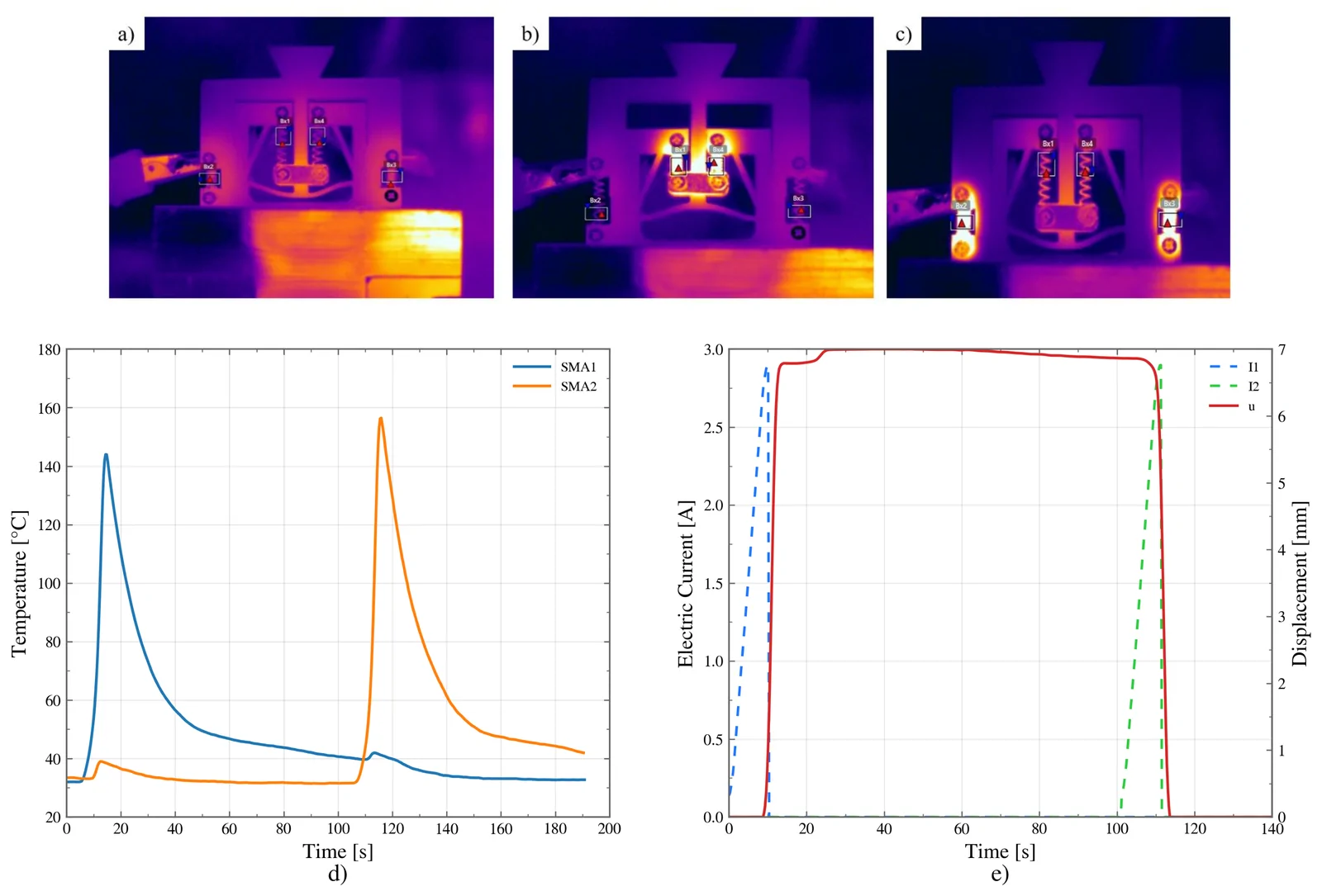
Actuator-level switching response during the exploratory upper-temperature test. (**a**–**c**) Representative infrared frames of the two antagonistic SMA branches. (**d**) SMA branch temperature histories, with peaks close to 160 degrees Celsius; these do not represent PETG body or TPU beam temperatures. (**e**) Applied branch currents and actuator displacement during one complete opening–closing cycle.

The actuator-level switching response is shown in Figure 13.

The measured displacement history shows a clear switching event associated with the bistable response. Starting from the first equilibrium state, the actuator reached a peak stroke slightly below 7 mm after the first current pulse and remained in the switched position during cooling, which confirms the passive-retention capability of the design. After the second pulse, the module returned to the original state. Thermal observations indicated peak SMA spring temperatures close to 160 degrees Celsius during this actuator-level experiment. This was an exploratory upper-temperature test under the slower activation protocol, not the operating temperature required for the subsequent integrated-panel switching sequence or a thermal qualification of the polymer components.

**Figure 14:**
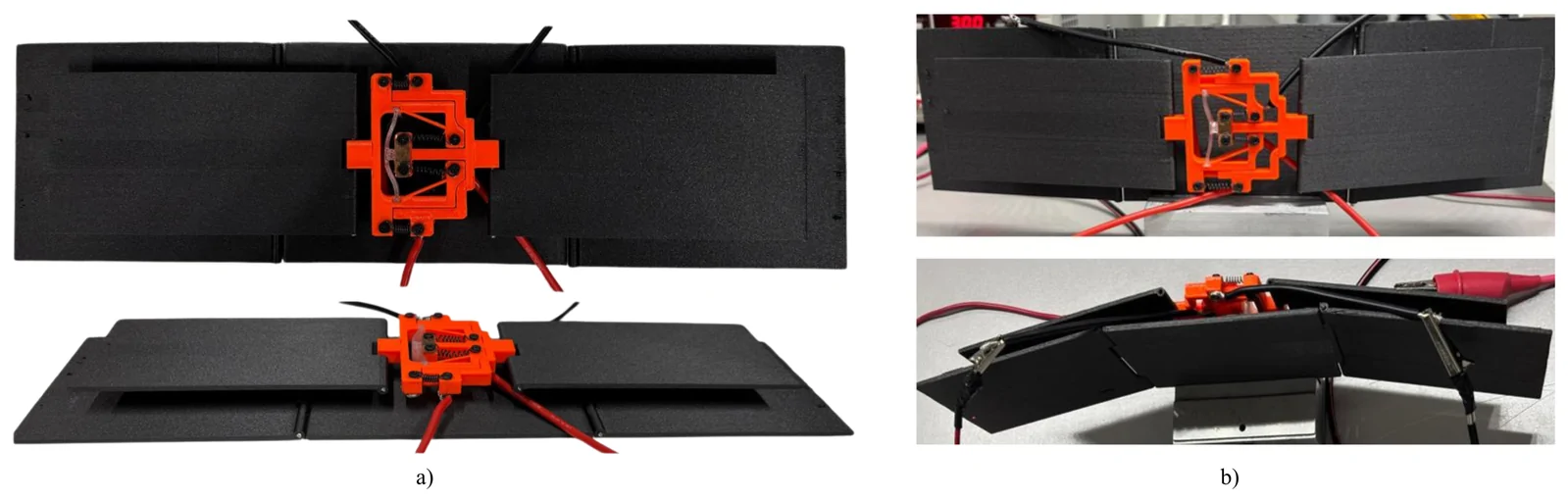
Integrated panel demonstrator in two mechanical configurations: (**a**) nearly flat and (**b**) curved after switching. These views document panel-level shape change, not complete-reflector surface accuracy or electromagnetic performance.

Under the panel-level protocol introduced above, the integrated demonstrator exhibited a measurable change in panel configuration, without establishing the accuracy of a complete reflector surface. Representative views of the integrated panel in the flat and deformed configurations are reported in Figure 14a,b. The observed edge displacement in the curved state is approximately 19 mm; this is a local mechanical output, not a spatial or ensemble mean of the reflector surface. The corresponding simulated value is 21.3 mm, so the experiment is lower by 10.8%. This displacement discrepancy cannot be assigned quantitatively to friction from the present measurements. Potential contributors include hinge and guide losses, fabrication and assembly tolerances, compliance of the load-transfer path, and differences between the measured point and the idealized kinematic output; their individual effects were not isolated. The panel motion model imposed the input displacement, so the output discrepancy alone cannot identify an equivalent friction force. The observed displacement supports panel-level mechanical switching and passive retention, not reflector-surface accuracy.

A single edge-displacement measurement does not uniquely determine the panel rotation, curvature radius, or spatial surface profile. Deriving these quantities by imposing the ideal simulated geometry would not provide independent experimental validation. No full-field surface measurements or surface RMS error relative to a prescribed reference surface are reported.

The repeatability sequence comprises ten complete opening–closing cycles of the tested demonstrator, not ten independent specimens or spatial surface samples. These repeated cycles document the switching function; cycle-resolved displacement statistics are unavailable, so statistical error bars are not assigned to the approximate 19 mm observation.

**Figure 15:**
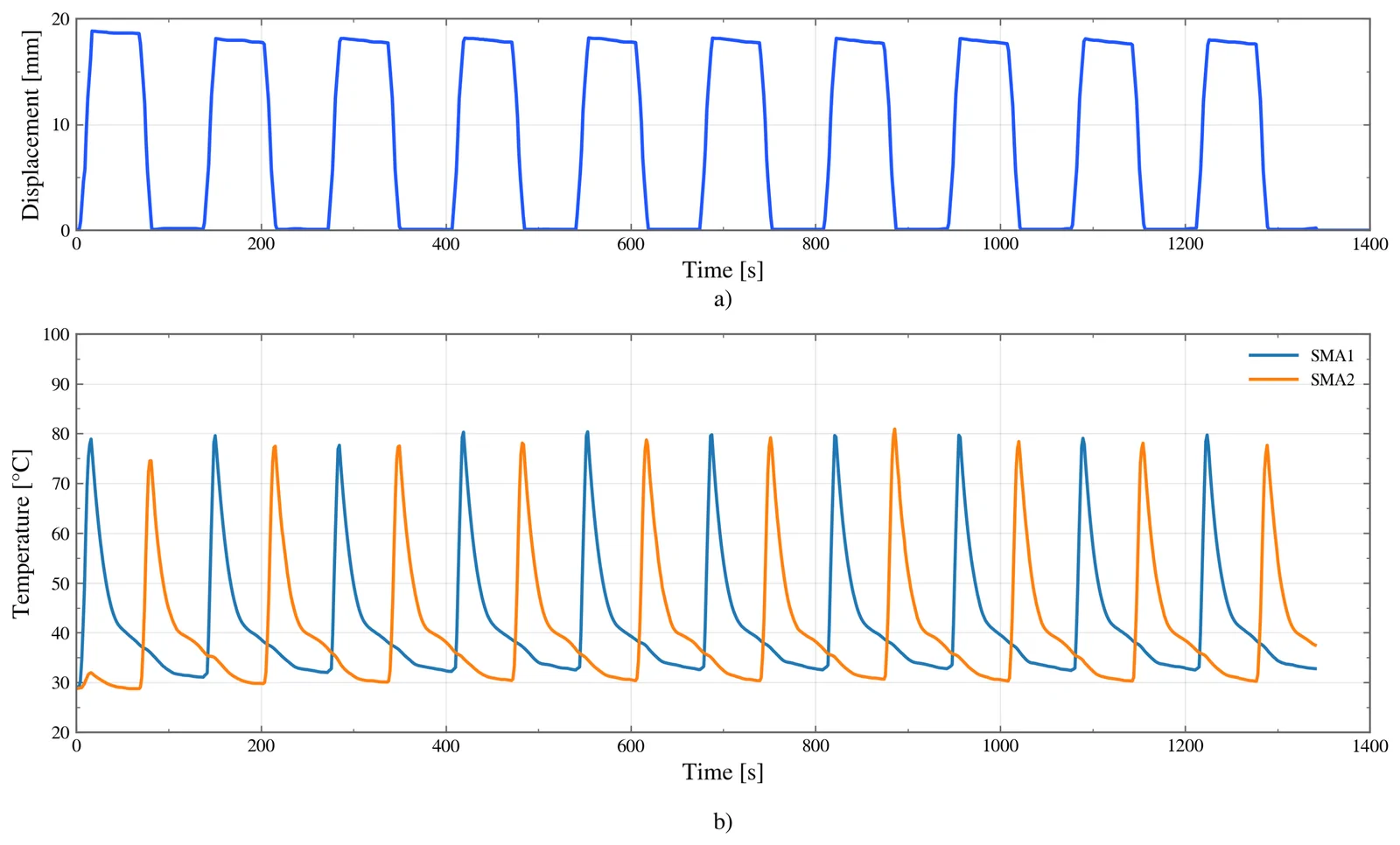
Panel-level repeatability and thermal response over ten complete switching cycles. (**a**) Edge displacement history during repeated opening–closing cycles. (**b**) Temperature histories of the two antagonistic SMA branches (SMA1 and SMA2), with peaks of approximately 80 degrees Celsius. The temperatures refer to the SMA branches, not to the reflector panels or actuator polymers.

Under the repeatability sequence introduced above, the available evidence supports a conservative conclusion: the module maintained stable switching behavior throughout the investigated sequence, with stroke values remaining approximately bounded between 0 mm and 19 mm. The displayed ten-cycle histories support a qualitative observation of short-term switching repeatability only. Cycle-resolved baseline means, standard deviations, and drift slopes were not quantified; no statistical bound on temperature or displacement drift is claimed. The ten-cycle displacement history and the alternating temperature peaks of the two SMA branches are shown in Figure 15a,b. This does not yet constitute a durability study, but it is sufficient to demonstrate short-term repeatability of the switching function. In the integrated-panel repeatability sequence, the SMA temperature peaks were approximately 80 degrees Celsius, as shown by the SMA1 and SMA2 histories in Figure 15b. These histories describe the SMA branches, not the Nylon-Carbon panels or the other polymer components.

The main experimental observations are summarized in Table 3.

### 4.3. Integrated Discussion and Limitations

The results position the proposed reconfiguration module as a mechanically validated subsystem for future CubeSat reflector architectures rather than as a complete antenna demonstrator. The experiments validate the switching principle, the integration of antagonistic SMA actuation with a compliant bistable core, and the ability of the module to induce measurable panel-edge displacement under electrically driven operation. This mechanical output is not a measurement of complete-reflector curvature, surface RMS error, or electromagnetic performance.

The main contribution is the integration of three features that are often treated separately in the literature: compact SMA actuation, passive bistable state retention, and explicit sizing for a CubeSat reflector-panel concept. Existing SMA-based antenna studies have already shown the feasibility of frequency, polarization, or geometry reconfiguration at component and subsystem level [18,21,22,23,24], while larger reflector-oriented demonstrations have highlighted the structural potential of SMA-enabled deployable antenna hardware [15,16]. In parallel, morphing-structure studies have shown that distributed SMA-driven shape adaptation is feasible in lightweight panels and adaptive composites [25], and compliant bistable mechanisms have been recognized as attractive for low-energy and space-relevant hardware [29,30]. The present study combines these elements into a single switching module supported by both actuator-level and panel-level experiments.

A second relevant outcome concerns the relationship between design intent and measured performance. The panel-level displacement of 19 mm, compared with a simulated value of 21.3 mm, indicates that the kinematic effect predicted numerically is substantially preserved in the physical demonstrator, albeit with a measurable penalty of 10.8%. This comparison supports the mechanical proof of concept, but does not identify the source of the displacement shortfall or establish that friction is negligible. Quantitative friction characterization and a force balance along the complete switching path remain limitations of the present validation.

The experiments also clarify the current maturity level of the system. The ten-cycle sequence demonstrates repeatable switching behavior over a short laboratory campaign, but it does not yet address high-cycle durability, environmental robustness, or property drift of the SMA elements and frictional interfaces. The electrical operating point supports a first-order switching-energy estimate, but not a complete system-level energy assessment or flight-oriented thermal optimization. The tests demonstrate functional switching, not assembly-level thermal qualification. TPU, PETG, and Nylon-Carbon temperatures were not measured separately, and brief SMA peaks do not imply equal polymer temperatures or establish creep damage. Local heating may affect stiffness; sustained-load creep and long-term stability remain uncharacterized.

SMA functional fatigue involves cycle-dependent evolution of recovery stroke, hysteresis, and transformation response, whereas structural fatigue involves damage accumulation leading to fracture [41]. Initial conditioning and long-term degradation must be distinguished: in the authors’ previous NiTi-wire study, repeated thermal cycling under constant stress led to stabilization mainly after 20–30 cycles (Section 2.1 and Figure 3 of Reference [35]). This wire-level result motivates preconditioning, but does not establish a universal stabilization threshold for the present springs. The 20 pre-installation conditioning cycles used here were intended to reduce initial transients; neither complete stabilization nor suppression of later transformation drift was quantified. Radiation-induced polymer changes and hinge/guide wear were not evaluated, and the ten-cycle assembled-module test demonstrates short-term repeatability, not service life.

PETG was used for laboratory prototyping. Bambu Lab PPS-CF and PET-CF are candidate rigid-body replacements, with reported HDT values of 235 and 182 degrees Celsius at 1.8 MPa, respectively. PET-CF reference specimens were annealed and dried at 80 degrees Celsius for 12 h. These specimen ratings do not qualify the assembly; replacement requires mechanical, electrical, and thermal checks and does not address TPU-core thermal sensitivity.

For the integrated-panel protocol, the peak electrical operating point gives a reference series-pair resistance of approximately 0.5 ohm. Assuming this resistance remains constant during the linear 3 s current ramp, integrating resistance times current squared gives an estimated 4.5 J per transition, or 9 J per opening–closing cycle. This is a first-order estimate at the SMA branch, not a voltage–current integral measured over the pulse; resistance changes during heating and transformation and control-system losses are not included. Both branches have zero commanded current during retention, so nominal SMA holding power is zero, whereas controller standby power was not measured.

Figure 15b shows peaks at approximately 80 degrees Celsius and a baseline near 30 degrees Celsius at the tested cadence, corresponding to a temperature rise of about 50 degrees Celsius. The approximately 60 s cooldown/retention interval pertains to these air tests. Different duty cycles were not investigated, so no duty-cycle-dependent temperature or energy map is claimed. The faster ramp is consistent with reduced heating duration compared with the exploratory actuator test, but the temperature comparison alone does not quantify energy savings or SMA degradation.

In vacuum, heat rejection relies on conduction and radiation rather than gas convection. Cooling and thermal crosstalk under vacuum were not assessed and cannot be inferred directly from these air tests.

Several limitations should therefore be stated explicitly:

- the validated output of the study is mechanical reconfiguration, not electromagnetic performance;
- the reflector panel was not characterized as a metallized radiating surface, so gain, beam shaping, and frequency-domain effects remain to be measured;
- the repeatability campaign is short-term and cannot be interpreted as a durability or qualification study; and
- the current prototype was tested in laboratory conditions rather than under representative space thermal, vacuum, vibration, or launch-load environments.

Taken together, these points define the correct scope of the study. The paper is best understood as a mechanics- and actuation-centered validation of a compact switching concept for future reconfigurable reflector systems. The next steps are metallization and electromagnetic testing of the reflector panel, more detailed electro-thermal power accounting, friction-aware design refinement, higher-cycle testing, and progressive validation under more representative environmental conditions.

## 5. Conclusions

The design separates SMA-driven switching from passive state retention: antagonistic springs trigger each transition, while the compliant bistable core maintains the selected configuration without continuous SMA heating. Integrating these functions in a compact module provides a mechanical proof of concept for reflector-panel reconfiguration within CubeSat geometric constraints.

The numerical work supports preliminary sizing, not mesh-independent predictive accuracy or independent validation of the isolated beam. The laboratory demonstration establishes short-term actuator- and panel-level switching, not complete-reflector accuracy, electromagnetic performance, durability, or space qualification. Further development should therefore prioritize thermal and mechanical characterization and reflector-level electromagnetic and environmental assessment, rather than extrapolating the present subsystem results to flight readiness.

## Figures and Tables

**Table 1:** Representative numerical cases used to establish the bistability logic of the curved beam.

Case	Geometry/Loading	Numerical Outcome	Interpretation
Case 1	h=5 mm, t=1.5 mm, Q=3.33, no pre-compression	Bistable response	Bistability in this simulated case; consistent with the classical constrained-beam reference.
Case 2	h=3 mm, t=1.5 mm, Q=2, no pre-compression	Monostable response	Loss of the second stable equilibrium in this simulated case, without establishing a universal threshold.
Case 3	h=3 mm, t=1.5 mm, Q=2, axial pre-compression 2.4 mm	Bistable response restored	Demonstrates the design value of pre-compression for compact beams.

**Table 2:** Final prototype parameters grouped by geometry, material model, and actuation settings.

Parameter Group	Value/Setup	Relevance to the Final Prototype
Target platform	3 U CubeSat envelope	Defines the packaging constraint that governs the module scale.
Reflector architecture	9 panels, approximately 83 mm×110 mm each	Sets the panel-level integration scale of the switching module.
Reference beam geometry for bistability study	h=5 mm, t=1.5 mm (Q=3.33); h=3 mm, t=1.5 mm (Q=2)	Provides the two benchmark geometries used to distinguish geometric bistability from monostability.
Representative axial pre-compression	about 2.4 mm for the compact Q=2 case	Restores bistability in the reduced-curvature configuration.
Beam material model in FE study	TPU, E≈39 MPa, ν=0.24	Defines the compliant-core constitutive assumptions used in ABAQUS.
Beam FE discretization	C3D20 elements, average mesh size about 0.6	Numerical setting used for the nonlinear bistability analyses.
Actuation architecture	Two antagonistic SMA spring pairs	Enables bidirectional switching without motorized transmission.
Passive retention element	Pre-curved, pre-compressed compliant beam	Maintains the stable state without continuous electrical input.
Actuator-level electrical input	Two current ramps up to 3 A, separated by approximately 100 s	Drives the opening and closing transitions of the stand-alone actuator.
Panel-level electrical input	Two current ramps up to 3 A, separated by about 61 s	Drives the integrated panel demonstrator between the two target shapes.
Target actuator stroke	about 7 mm	Mechanical output used for module sizing and reflector kinematic input.
Imposed reflector-side displacement	about 3.5 mm per side	Symmetric input applied in the reflector motion model.

**Table 3:** Key experimental results for the proposed reconfiguration module.

Test Level	Quantitative Outcome	Interpretation
Complete actuator	Peak stroke slightly below 7 mm	Confirms bistable switching of the SMA-driven module.
Complete actuator	Peak SMA spring temperature about 160 degrees Celsius	Exploratory upper-temperature test under the slower activation protocol; not a measured polymer temperature or a qualified operating limit.
Integrated panel	Approximate edge displacement 19 mm	Local mechanical switching output; not a measurement of curvature radius, panel angle or surface RMS error.
Panel experiment vs. simulation	19 mm measured vs. 21.3 mm simulated	Displacement is 10.8 percent lower than simulation; individual contributions from friction and other model–prototype differences were not isolated.
Repeatability sequence	10 full cycles in about 1340 s	Supports stable short-term switching behavior under the tested laboratory conditions.
Integrated panel thermal response	Peak SMA spring temperature about 80 degrees Celsius	Repeated panel switching under the investigated laboratory protocol; no local polymer temperatures or long-term thermal limits established.

## Data Availability

The original contributions presented in this study are included in the article. Further inquiries can be directed to the corresponding author.
